# Incorporation of non-natural nucleotides into template-switching oligonucleotides reduces background and improves cDNA synthesis from very small RNA samples

**DOI:** 10.1186/1471-2164-11-413

**Published:** 2010-07-02

**Authors:** Jeremy Kapteyn, Ruifeng He, Eric T McDowell, David R Gang

**Affiliations:** 1 School of Plant Sciences and BIO5 Institute, The University of Arizona, 1657 E Helen Street, Tucson, AZ 85721, USA; 2 Institute of Biological Chemistry, Washington State University, PO Box 646340, Pullman, WA 99163, USA

## Abstract

**Background:**

The template switching PCR (TS-PCR) method of cDNA synthesis represents one of the most straightforward approaches to generating full length cDNA for sequencing efforts. However, when applied to very small RNA samples, such as those obtained from tens or hundreds of cells, this approach leads to high background and low cDNA yield due to concatamerization of the TS oligo.

**Results:**

In this study, we describe the application of nucleotide isomers that form non-standard base pairs in the template switching oligo to prevent background cDNA synthesis. When such bases are added to the 5' end of the template switching (TS) oligo, they inhibit MMLV-RT from extending the cDNA beyond the TS oligo, thus increasing cDNA yield by reducing formation of concatamers of the TS oligo that are the source of significant background.

**Conclusions:**

Our results demonstrate that this novel approach for cDNA synthesis has valuable utility for application of ultra-high throughput technologies, such as whole transcriptome sequencing using 454 technology, to very small biological samples comprised of tens of cells as might be obtained via approaches like laser microdissection.

## Background

Methods such as laser (capture) microdissection [1] and cell sorting are becoming increasingly accessible, enabling more researchers to address biological questions at the level of individual cell types. As the sensitivity of analytical tools such as mass spectrometry increases, very small samples become increasingly amenable to systems-level global analyses including proteomics and metabolomics investigations. At the same time, continually decreasing costs of next-generation sequencing technologies, combined with the added qualitative information value of sequence data [2-5] as compared to microarrays, could make transcriptome sequencing the preferred means of evaluating questions regarding gene expression and alternative splicing for model organisms, and *de novo *sequencing continues to represent the best approach for non-model organisms where genomic sequence data is lacking. The ability to conduct omics level experiments using only a few cells will allow researchers to answer questions that were previously intractable due to the heterogeneous and complex nature of most tissue samples.

Despite the growing availability of next-generation sequencing technologies, significant effort is still often needed to collect the required biological starting material. For transcriptome analysis, this generally entails the synthesis and subsequent amplification of cDNA. Although exponential amplification raises concerns about representation bias, exponentially amplified cDNA is representative of the transcript population, even when starting with the equivalent of a single cell's RNA [6,7]. Moreover, full-length transcripts are highly desirable in transcriptome sequencing experiments. Several methods have been developed to preferentially enrich cDNA for full length transcripts using the 5' cap structure of mature mRNA transcripts [8-12]. Among these is the template switching PCR (TS-PCR) method [12], which takes advantage of the terminal transferase activity and template-switching ability of Moloney murine leukemia virus reverse transcriptase (MMLV-RT) to add an arbitrary sequence to the 5' end of a transcript in a manner that occurs preferentially for capped, full-length transcripts [12-16]. This arbitrary sequence, encoded in a TS oligo, along with a second arbitrary sequence added at the 3' end of the cDNA by the oligo-dT-containing primer used to initiate reverse transcription, is used to subsequently amplify the total pool of putatively full-length transcripts in a completely sequence-naïve manner. For investigators interested in transcriptome analysis and gene discovery from non-model organisms or those interested in transcriptional processes at a cell-specific level, TS-PCR cDNA synthesis represents the most straightforward approach to generating cDNA for sequencing efforts. However, this approach does suffer from some drawbacks, including the potential for high background that interferes with downstream sequencing efficiencies when starting with very small quantities of RNA.

**Figure 1:**
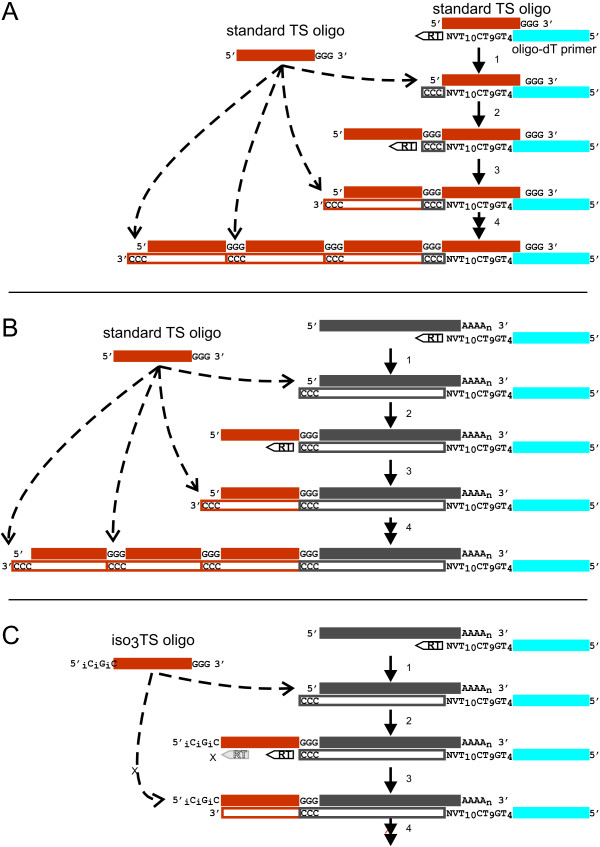
**Model of cDNA synthesis that occurs in the absence (A) and presence (B and C) of RNA using standard TS oligo (A and B) vs. iso_3_TS oligo (C)**.

In this study, we describe a novel approach to eliminate background and increase cDNA yield in TS-PCR. The key feature of our modification that enables this improvement of the TS-PCR method is the inclusion of isomeric nucleotide bases at the 5' end of the TS oligo to inhibit MMLV-RT template switching activity after incorporation of the first TS oligo and thereby reduce or prevent TS oligo concatamerization (see Figure 1). We applied the modified method to construct cDNA libraries from glandular trichome secretory cells of tomato (*Solanum*) species and petunia, as well as from rhizome tips and elongation zones of scouring rush (*Equisetum hyemale*) and red rice (*Oryza longistaminata*). Our results demonstrate that application of the improved TS-PCR method dramatically reduced the proportion of oligo concatamers in the resulting sequence data, and the cDNAs produced were of high quality and yield, useful for both conventional Sanger sequencing as well as next-generation sequencing.

## Results

Based on results presented below, we developed the following model that outlines how cDNA synthesis occurs in the absence and presence of RNA using standard TS oligo vs. iso_3_TS oligo (Figure 1). When a total RNA sample including mRNA transcripts is combined with oligo-dT primer sequence to prime first strand synthesis in the presence of TS oligo, the following can occur. **Step 1 **- Oligo-dT primer anneals to the polyA tail of an mRNA transcript (Figure 1B) and reverse transcriptase (RT) synthesizes first strand cDNA, or to some other polynucleotide entity in solution (Figure 1A), such as a standard TS oligo. Upon reaching the 5' end of the transcript (or other nucleotide entity), the terminal transferase activity of MMLV RT adds several additional bases, preferentially dC, to the 3' end of the cDNA strand. The terminal transferase activity of MMLV-RT is greatly enhanced in the presence of Mn^2+ ^[17]. **Step 2 **- The rG_3 _bases at the 3' end of a standard TS oligo anneal to the polyC bases left by the RT at the 3' end of the cDNA strand and RT switches templates, transcribing through the TS oligo DNA template (Figure 1A and 1B). The TS oligo sequence and the extended 5' portion of the oligo-dT primer are used as primer sites for cDNA amplification in the LD PCR step which follows the RT reaction. **Step 3 **- The terminal transferase activity of RT can add polyC bases to the end of a cDNA strand when using the standard TS oligo DNA as a template (Figure 1A and 1B). **4 **- Another TS oligo can then anneal and the process of TS oligo transcription and addition can continue indefinitely, adding a series of direct repeats of TS oligo sequence to the growing cDNA strand. **Alterative Step 3 **(iso_3_TS oligo is used) - Upon reaching the poly iso-nucleotide bases at the 5' end of the iso_3_TS oligo, RT is inhibited from incorporating the corresponding nucleotides and reverse transcription terminates without addition of a polyC tail (Figure 1C). **Alterative Step 4 **- No additional iso_3_TS oligo can anneal and concatamerization of TS oligo does not occur (Figure 1C).

**Figure 2:**
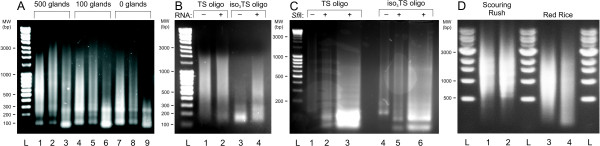
**Analysis of products formed in RT reactions with or without the use of modified TS oligos**. (**A**) **Comparison of background amplification between small biological samples of different sizes and negative control using standard TS oligo**. cDNA synthesis for different quantities of starting material after TS-PCR (lanes 1, 4 and 7). *Sfi*I digestion of negative control (lane 9) and samples representing 500 and 100 tomato glands (lanes 3, 6). Lanes 2, 5, and 8 are mock restriction digestions without enzyme. (**B**) **Comparison of cDNA synthesis performed using TS oligo vs. iso_3_TS oligo**. Negative control cDNA synthesis reactions without RNA but with standard TS oligo (lane 1) or with iso_3_TS oligo (lane 3). cDNA synthesis with tomato gland RNA sample using TS oligo (lane 2) and iso_3_TS oligo (lane 4). (**C**) **Comparison of *Sfi*I digested tomato glandular trichome cDNA after TS-PCR cDNA synthesis using standard TS oligo vs. iso_3_TS oligo**. Tomato gland cDNA synthesized using TS oligo (lane 1) and after *Sfi*I digestion (lanes 2 and 3), or using iso_3_TS oligo (lane 4) and digested with *Sfi*I (lanes 5 and 6). (**D**) **Size distribution and concentration of cDNA samples produced using the iso_3_TS oligo with larger RNA samples**. Lane 1: cDNA from rhizome tips of scouring rush (*Equisetum hymale*); lane 2: cDNA from rhizome elongation zones of scouring rush; lane 3: cDNA from rhizome tips of red rice (*Oryza longistaminata*); lane 4: cDNA from rhizome elongation zones of red rice; L: 500 ng of 1 kb DNA ladder (NEB).

We developed this model as the result of performing experiments where we used TS-PCR to construct cell-type specific cDNA libraries from small numbers of isolated glandular trichome secretory cells from tomato and related *Solanum *species. These species contain several different types of glandular trichomes, some of which can only be isolated from other types by hand-selecting them from the surfaces of fresh and intact plant leaves using microcapillary probes under the guidance of a stereoscope, a time consuming and tedious procedure that yields only small numbers of isolated trichomes (100 and 500 gland samples were evaluated, see Figure 2). While constructing libraries from these very small samples, we encountered a significant flaw in the TS-PCR method as originally described [12]: a substantial quantity of cDNA spanning a size range that might be expected to represent a typical biological sample is synthesized in the absence of any starting RNA (Figure 2A, lane 7; 2B, lane 1; 2C, lane 1; see Figure 1A). Differences in level of cDNA synthesis for different quantities of starting material are difficult to distinguish after TS-PCR (Figure 2A, lanes 1, 4 and 7). Substantial cDNA is generated in TS-PCR step even for negative control (Figure 2A, lane 7). However, after *Sfi*I digestion, the cDNA in the negative control is digested into low MW fragments (Figure 2A, lane 9), while a smear of higher MW DNA is present for samples representing 500 and 100 tomato glandular trichomes ("glands") (Figure 2A, lanes 3, 6). Lanes 2, 5, and 8 are mock restriction digestions without enzyme added.

Negative control cDNA synthesis reactions performed without RNA show high levels of cDNA synthesis for TS oligo as large as 2000 bp (Figure 2B, lane 1), while the negative control cDNA synthesis performed using iso_3_TS oligo demonstrates almost complete suppression of artefactual synthesis of cDNA above 200 bp (Figure 2B, lane 3). It is difficult to distinguish the results of cDNA synthesis for the negative control vs. tomato gland RNA sample using TS oligo (Figure 2B, lane 1 vs. 2), while the results of cDNA synthesis using iso_3_TS oligo for the negative control (Figure 2B, lane 3) and the tomato gland RNA sample (Figure 2B, lane 4) show strong dependence on the presence of sample RNA to generate cDNA.

After preparative DNA electrophoresis, tomato gland cDNA synthesized using TS oligo (Figure 2C, lane 1) shows a stronger shift toward lower molecular weight fragments after *Sfi*I digestion (Figure 2C, lanes 2 and 3) than the cDNA synthesized from the same RNA sample amplified using iso_3_TS oligo (Figure 2C, lane 4) and digested in parallel with *Sfi*I (Figure 2C, lanes 5 and 6). Lanes 3 and 6 were large preparative lanes used for subsequent cDNA library production as described in the Methods. Equal quantities of DNA were loaded in lanes 1, 2, 4, and 5 and in 3 and 6, but the proportion of the DNA sample comprising lower molecular weight fragments as evidenced by ethidium bromide staining is much higher after *Sfi*I digestion for samples synthesized using the unmodified TS oligo (Figure 2C, lanes 2 and 3) as compared to that for cDNA synthesized using the modified, isoTS oligo (Figure 2C, lanes 5 and 6). This suggests that a higher proportion of cDNA synthesized with the unmodified TS oligo consists of concatamers of the TS oligo.

**Figure 3:**
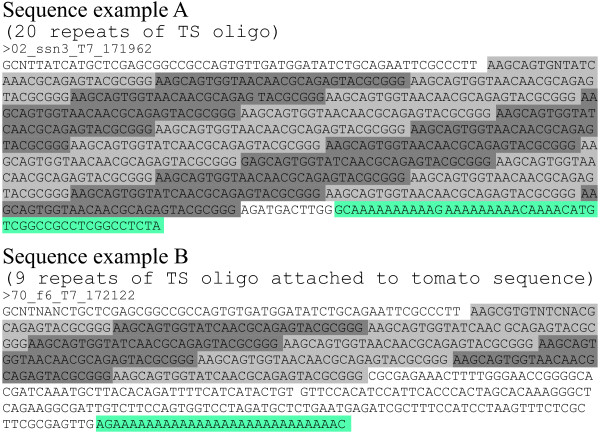
**Examples of sequencing results for cDNA clones having concatamers of TS oligo**. Sequence example A reflects a cDNA clone with an insert that appears to be comprised entirely of a concatamer of 20 direct repeats of TS oligo (highlighted in alternate shades of gray) associated with a single oligo-dT reverse primer (highlighted in turquoise). The 11 intervening bases are of unknown origin. See Figure 1A for a diagrammatic representation of the process whereby this type of concatamer may arise. Sequence example B shows 9 repeats of TS oligo concatamerized to a 178 base sequence with homology to nucleotide sequences from *Vitus vinifera *and *Arabidopsis thaliana *potentially representing a plant metalloendopeptidase. The oligo-dT reverse primer is also shown. See Figure 1B for a representation of the mechanism by which this type of concatameric sequence would be generated.

To characterize the sequences of the apparently artefactual cDNA generated using TS oligo in first strand cDNA synthesis reactions, performed in the absence of RNA or using very small quantities of sample RNA, we cloned amplified and undigested cDNA into pCR2.1-TOPO and sequenced several cDNA inserts, revealing them to be concatamers of direct repeats of the TS oligo (Figure 3A). This concatamerization of TS oligo occurs in cDNA syntheses from biological samples containing very small amounts of RNA (Figure 2A, lanes 4-6), but appears to be reduced as the size of the biological sample increases (Figure 2A, lanes 1-3). In samples containing either no RNA or very low numbers of mRNAs to serve as template for reverse transcription, RT activity is spuriously primed via primer-primer annealing (Figure 1A). Although the efficiency of MMLV-RT terminal transferase activity toward uncapped RNA templates under the conditions used has been shown to be much lower than for capped transcripts (activity with DNA templates was not examined) [17], the low abundance of transcript templates increases the likelihood of terminal transferase addition of dC residues to the growing cDNA strand at the ends of any available template, including non-capped templates. After MMLV-RT has switched templates to the DNA TS oligo, the process is repeated and TS oligo is added indeterminately to the 3' end of the growing cDNA strand (see Figures 1A/B and 3).

This background cDNA synthesis in the absence of RNA or in the presence of low amounts of RNA and approaches to eliminate it were not mentioned in the original or subsequent publications on the TS method [12]. The original TS patent (U.S Pat. No. 5,962,272) mentions a background problem, but does not describe or speculate as to its cause. However, this problem presents a serious obstacle to effective construction of cDNA libraries representing biological transcripts rather than synthesis artefacts, especially for very small RNA samples originating from just a few cells. One approach to circumvent this problem is to incorporate restriction endonuclease sites into the oligos used during cDNA synthesis. This facilitates ligation into cloning vectors for library construction and reduces concatamerization artefact contamination of the final cDNA sample. However, cDNA size fractionation does not eliminate all of the small cleaved fragments and they contaminate the final cDNA library, resulting in a lower proportion of transcript-based cDNAs available for ligation into a cloning vector (Figure 2A, lanes 3,6). Moreover, overall yield of high quality cDNA representing biological transcripts is also lower when the reverse transcriptase spends a significant proportion of its time synthesizing concatamers as opposed to unique cDNAs.

We have developed a unique method to eliminate this concatamer-generated background problem that takes advantage of the expanded genetic alphabet [18,19] to prevent MMLV-RT from reaching the 5' end of the TS oligo template after the initial template switch, thus preventing terminal transferase-mediated addition of dC bases at the end of the sequence complementary to the TS oligo template and subsequent concatamerization of TS oligo sequences (Figure 1C). Stereoisomers of deoxycytosine and deoxyguanosine, referred to as isocytosine (iso-C) and isoguanosine,(iso-G), have the properties of natural nucleotide bases but have altered hydrogen bonding abilities which cause them to form nonstandard base pairs specifically with each other and discriminate against their naturally occurring analogs [17,19]. Until now, application of this expanded alphabet has been extended only to hybridization-based molecular diagnostic assays [20].

We designed a modified TS oligo (called iso_3_TS, see Materials and Methods) that incorporated iso-C and iso-G bases at the 5' end of the oligo to test their ability to inhibit the MMLV-RT terminal transferase activity in a first strand synthesis mix that lacked the complementary iso-dNTPs. We designed a series of three isomeric nucleotide bases to discourage any potential infidelity that MMLV-RT might exhibit. The performance of the iso_3_TS oligo in TS-PCR cDNA synthesis was tested in parallel with an identical TS oligo lacking the isomeric bases to determine the effect of the modified oligo on the production of artefactual cDNA in both negative control cDNA synthesis having no RNA present in the reverse transcription step as well as a test RNA sample isolated from a small number of tomato glandular trichomes. As shown in Figure 2B, performing cDNA synthesis using the iso_3_TS oligo greatly reduces or eliminates artefactual cDNA that resulted from concatamer formation and amplification in the cDNA synthesis, as occurred when using the unmodified TS oligo either with a small quantity of RNA template or without RNA template. It was difficult to distinguish between the intensity or size range of artefactual cDNA synthesized in the latter two cases, suggesting that a large proportion of the cDNA synthesized for the biological sample may consist of artefactual cDNAs while a smaller proportion of the cDNA population represents actual transcripts. This conclusion was supported by the results of subsequent *Sfi*I digestion of the cDNA samples in preparation for their ligation into the library cloning vector (Figure 2C), where cDNA samples prepared using unmodified oligo showed a strong shift toward lower molecular weight fragments after restriction digestion, a shift that was not evident for cDNA synthesized using iso_3_TS oligo.

Evidence of the efficacy of the iso_3_TS oligo can be observed in comparisons of several of the EST libraries we have recently made and sequenced using 454 technology. For instance, in the *Salvia divinorium*, *Solanum lycopsericum*, and *Solanum habrochaites *glandular trichome libraries, we found that 20.6%, 17.9%, and 11.6% of the reads, respectively, contained 2 or more concatamers of the SMART IV-derived oligo (TS oligo). Many of these reads contained multiple concatamers as illustrated in Figure 3. This number of concatamers is dramatically reduced or eliminated via the use of the iso_3_TS oligo as seen in the *Equisetum hyemale*, *Solanum habrochaites *and *Isatis tinctoria *454 libraries which possess 0.02%, 1.5%, 3.1% of reads possessing 2 or more concatamers of the SMART IV-derived oligo (TS oligo) (Table 1), with the vast majority of these possessing only 2 concatamers. In all of these cases, the same method for cDNA synthesis, 454 library construction and sequencing was used, and involved the same amount of template RNA. With the modified method, we also produced high quality cDNA from rhizome tips and elongation zones of scouring rush and red rice that was suitable for next generation sequencing using the 454 GS-FLX instrument with Titanium technology (Figure 2D).

**Table 1:** Comparison of sequencing data from different cDNA libraries generated by standard TS oligo vs. iso_3_TS oligo

cDNA synthesis method	Species	Avg length of read (nt)	Num. 454 reads (Mb)	Num. 2+ SMART oligos (concatamers)	% of concatamers
Standard TS oligo	*Salvia divinorium*	197	342263 (77.6)	70549	20.6
	*Solanum lycopsericum*	207	336745 (70.0)	60173	17.9
	*Solanum habrochaites*	165	329070 (54.4)	38231	11.6

Modified TS oligo (iso_3_TS oligo)	*Solanum habrochaites*	199	344190 (68.6)	5032	1.5
	*Isatis tinctoria*	345	970893 (335.9)*	29734	3.1
	*Equisetum hyemale*	324	871579 (282.2)*	159	0.02

* data from 454 Titanium sequencing run

## Discussion

The use of oligos containing isomeric nucleotide bases to inhibit the enzymatic activity of MMLV-RT at a discrete point in reverse transcription of a template represents a novel application of the expanded genetic alphabet. The use of this approach to modify standard TS-PCR cDNA synthesis methods permits elimination of TS oligo concatamer formation, dramatically changing the composition of the first strand cDNA population used as template for the LD PCR step in TS-PCR based cDNA synthesis by removing a huge proportion of sequence comprised only of direct repeats of the TS oligo. Amplification of these concatamers in the PCR step of the TS-PCR generates a substantial quantity of artefactual sequence in the amplified population of cDNA. Upon digestion, TS oligo fragments can potentially represent a vast molar majority of the DNA fragments in the cDNA population, making downstream cloning and sequencing inefficient and more costly. Reduction or elimination of TS concatamer formation via use of the iso_3_TS oligo and the resultant increase in the efficiency of cDNA synthesis from the biological sample itself increases the signal to noise ratio and restores the biological integrity of cDNA samples synthesized from very limited samples.

Performing first strand cDNA synthesis using the iso_3_TS oligo while providing only the four naturally occurring nucleotide bases apparently causes MMLV-RT to prematurely stall before reaching the 5' end of the iso_3_TS oligo template (see Figure 1). The great reduction of concatamer formation in these reactions suggested that misincorporation of natural bases by MMLV-RT opposite the synthetic bases was inhibited and that MMLV-RT terminal transferase activity was reduced, although we did not measure actual terminal transferase activity. However, we believe that our data only support a mechanism for concatamer formation that involves the terminal transferase activity of MMLV-RT. Because the "monomers" are all added in the same orientation, a mechanism involving addition of primer-dimers is precluded. The only other potential mechanism for concatamer formation might involve blunt ligation of single stranded TS oligos to the end of the template followed by replication by MMLV-RT. However, no enzyme that could perform such a ligation was present in the assays. Moreover, the elimination or dramatic reduction in concatamer formation that was observed when the iso_3_TS oligo was used but not when the standard TS oligo was used fully supports our proposed mechanism. The fact that some concatamers (almost exclusively just single concatamer additions) were observed when the iso_3_TS oligo was used suggests that a residual level of terminal transferase activity was still present. However, our results clearly show that this activity was greatly reduced when compared to conditions using the original TS oligo. Perhaps using more than three iso-C/iso-G bases and/or addition of Mn^2+ ^ions would help reduce this activity further as the presence of Mn^2+ ^appears to have a very dramatic effect on terminal transferase activity and generation of CCC tail [17].

The increased efficiency of TS-PCR achieved by use of the iso_3_TS oligo design enhances the amenability of small or rare biological samples to genomics investigation and may facilitate comparative functional genomics approaches to understanding processes such as cancer pathogenesis or stem cell differentiation at very early stages. Very small biological samples, on the order of 10 s to 100 s of cells, would be sufficient to generate cDNA for cloning and sequencing, potentially opening new avenues for investigation of important biological processes.

## Conclusions

The procedure presented here was developed to permit synthesis of cDNA using the TS-PCR approach from very small quantities of starting material, such as manually selected glandular trichomes or any sample of a particularly limited nature. cDNAs are synthesized using an adaptation of the SuperSMART approach relative to the sample handling. However, the primers used are similar to those from the SMART cDNA synthesis kit to enable *Sfi*I digestion of the cDNA and ligation into the pDNR-LIB vector for library propagation in *E. coli *and subsequent Sanger sequencing. Digestion with *Sfi*I also allows for cDNA produced in this way have most of the primer sequence removed prior to use in massively parallel sequencing approaches (next-generation sequencing) such as 454 Life Sciences GS FLX Titanium series sequencing. Validation of the utility of this method in such approaches was provided by successful construction and 454 sequencing of cDNA from rhizome samples of two divergent plant species: scouring rush (fern ally, "primitive plant") and red rice (monocot, "advanced plant"). We have continued to use this method to produce cDNA samples from several other plant species with similarly successful results in next-gen sequencing.

## Methods

### Tissue collection

Glandular trichomes (glands) were selected by hand from intact leaves of various *Solanum *and other species. Briefly, very fine, stretched glass microcapillaries were drawn by hand and the ends sealed. These glass microcapillary probes were subsequently used to touch and remove specific types of glandular trichomes from the surfaces of the leaves. A counted number of glands was collected on a probe over a short period of time, and the end of the probe with the glands was transferred to a microfuge tube containing 100 μl of RNAlater (Ambion, Austin, TX, USA). RNA isolation was performed after the desired number of glands had been accumulated (see Results for number of glands collected for specific experiments). Rhizome tips and elongation zones (~10 - 100 mg tissue) were dissected and collected from fresh rhizomes of rhizomatous species, scouring rush and red rice, and immediately frozen in liquid nitrogen.

### RNA isolation

RNA was isolated from glandular trichome samples using Machery-Nagel (Clontech, Mountain View, CA) NucleoSpin RNA II kits, following the manufacturer's instructions, with the following exceptions:1) 350 μl of buffer RA1 (with B-mercaptoethanol added) was added to manually selected glands contained in microfuge tube in 100 μl of RNAlater (Ambion); 2) glands were disrupted using a probe sonicator (Branson Sonifier 450, setting #4 for ~30 s using microprobe); 3) RNA elution was done in 2 steps, using 2 volumes of 27 μl of nuclease-free water, into the same microfuge tube, yielding a total eluate volume of slightly more than the 52.5 μl for use as input for the first strand cDNA synthesis step below. For rhizomatous species, RNA was isolated from rhizome tips and elongation zones using RNeasy Plant Mini Kit (Qiagen, Valencia, CA) according to the manufacturer's instructions. These different RNA isolation kits were used to ensure that RNA isolated by different approaches would work equally well in the modified cDNA synthesis procedure outlined below.

### Primer sequences

Primers for first strand synthesis: TS oligo (*Sfi*I site **bold**) 5'-AAGCAGTGGTATCAACGCAGAGT**GGCCATTACGGCC**rGrGrG-3' (rG = ribonucleotide G); iso_3_TS oligo (*Sfi*I site **bold**) 5'-iCiGiCAAGCAGTGGTATCAACGCAGAGT**GGCCATTACGGCC**rGrGrG-3' (iC = iso-dC, iG = iso-dG); Oligo-dT primer (*Sfi*I site **bold**) 5'-TAGA**GGCCGAGGCGGCC**GACATGTTTTGTTTTTTTTTCTTTTTTTTTTVN-3'. Primers for LD-PCR: iso_3_TS oligo as 5'PCR primer; 3'PCR primer: 5'-TAGAGGCCGAGGCGGCCGAC-3'.

### First strand cDNA synthesis

First strand cDNA synthesis was performed in 106 μl reactions assembled in the following manner. For each sample, the following components were placed in a 200 μl PCR tube on ice: 52.5 μl RNA sample, 8.4 μl oligo dT primer (10 μM), 8.4 μl TS oligo or iso_3_TS oligo (10 μM), 69.3 μl total volume. RNA and primer mixes were denatured at 65°C for 2 min and then removed to ice. The following reagents were then added to each reaction tube: 20 μl 5× first strand buffer (Powerscript [Clontech] RT buffer: 250 mM Tris (pH 8.3), 30 mM MgCl_2_, 375 mM KCl, 20 mM DTT), 2 μl DTT (100 mM), 10 μl 50× dNTP (10 mM), 5 μl SuperScript II (Invitrogen, Carlsbad, CA). First strand reactions were incubated at 42°C for 2 hr.

### First strand cDNA cleanup

A cleanup step was performed for first strand cDNA samples using Machery-Nagel NucleoSpin Extract II kits following the manufacturer's protocol and the total recovered elution volume was 80-85 μl per sample.

### cDNA amplification by Long-Distance PCR (LD PCR)

For PCR amplification of full length cDNAs, 80 μl of cleaned-up first strand cDNA was used as template in 100 μl PCR reactions including the following reagents: 1× Advantage 2 PCR Buffer (Clontech), 0.2 mM dNTPs, 0.12 μM 5'PCR primer (iso_3_TS oligo), 0.12 μM 3'PCR primer, and 1× Advantage 2 Polymerase Mix. PCR amplifications were performed using an MJ Research Dyad (MJ Research, Waltham, MA, USA) with the following program parameters: an initial hold at 95°C for 1 min, followed by 27 cycles of 95°C for 5 s, 65°C for 5 s, and 68°C for 6 min. After completion of a PCR reaction, a 5 μl aliquot of each sample was removed and run on a 1.1% agarose/TAE gel containing 0.5 μg/ml ethidium bromide alongside a DNA MW marker such as the Fermentas Mass Ruler (Fermentas, Glen Burnie, MA, USA).

### LD PCR reaction clean up

LD PCR amplified cDNA samples were cleaned up using NucleoSpin II kits following the manufacturer's protocol and the total combined eluate was approximately 32 μl.

### *Sfi*I digestion of cDNA

*Sfi*I digestion was performed in 40.5 μl total volumes containing 32 μl cDNA, 4.05 μl 10× *Sfi*I buffer 4.05 μl *Sfi*I enzyme, and 0.4 μl 100× BSA. Digestions were incubated at 50°C for 2 hrs before proceeding to cDNA size fractionation. *Sfi*I buffer and enzyme were obtained from NEB (Ipswich, MA, USA).

### cDNA size fractionation by gel electrophoresis

cDNA was fractionated by molecular weight using preparative DNA gel electrophoresis after *Sfi*I digestion. Digested cDNA samples were loaded (using a loading buffer containing bromophenol blue) on a 2% low melt agarose (NuSieve GTG, Cambrex, Rockland, ME, USA) gel containing 0.5 μg/ml ethidium bromide. Electrophoresis was performed at a low voltage setting (5-8 V/cm) until the bromophenol blue dye had migrated approximately 4 cm. Gels were photographed and the lanes excised from the 400 bp molecular weight marker up to the sample well. DNA was isolated from the gel slice using a Qiagen MinElute kit (Valencia, CA, USA) and 6 sample volumes of buffer QG. Elution was performed using 10 μl of elution buffer. Ligations into pDNR-LIB (Clontech) were performed immediately following cDNA size fractionation and elution according to the method specified in the SMART cDNA library construction manual. Sequencing of gland-derived samples was accomplished using traditional Sanger sequencing of individual clones. The digested, fractionated cDNA samples from rhizome tips and elongation zones were purified using QIAquick PCR purification kit (Qiagen). 5~10 μg purified cDNA from each rhizome sample was used in construction of a 454 Genome Sequencer FLX [21-23] fragment library using Titanium series reagents http://www.454.com followed by sequencing on a GS FLX instrument.

## Authors' contributions

JK and RH carried out the experiments, analyzed the data and wrote the manuscript. ETM participated in sample collection and library preparation. DRG designed and coordinated the study, and edited the manuscript. All authors read and approved the final manuscript.
